# Retraction Note: Effect of rosuvastatin on fasting and postprandial endothelial biomarker levels and microvascular reactivity in patients with type 2 diabetes and dyslipidemia: a preliminary report

**DOI:** 10.1186/s12933-019-0841-1

**Published:** 2019-03-16

**Authors:** Kyoung Min Kim, Kyong Yeun Jung, Han Mi Yun, Seo Young Lee, Tae Jung Oh, Hak Chul Jang, Soo Lim

**Affiliations:** 1 Department of Internal Medicine, Seoul National University College of Medicine and Seoul National University Bundang Hospital, 82 Gumi-ro 173beon-gil, Bundang-gu, Seongnam, 463-707 South Korea; 2 Department of Internal Medicine, Eulji General Hospital, Seoul, South Korea; 3 Physiologic Diagnostic Laboratory, Vascular Laboratory, Seoul National University Bundang Hospital, Seongnam, South Korea

## Retraction to: Cardiovasc Diabetol (2017) 16:146 10.1186/s12933-017-0629-0

The authors have retracted this article [1] because they have identified serious errors in their data analysis which change the conclusions of their study. All authors agree with this retraction.
